# Effect of dietary supplementation of fermented fish silage on serum biochemical parameters of broiler Japanese quails (*Coturnix coturnix japonica*)

**DOI:** 10.14202/vetworld.2017.380-385

**Published:** 2017-04-06

**Authors:** Sasmita Panda, Laxman Kumar Babu, Arun Kumar Panda, Tanuja S, Anurag Mohanty, Kuldeep Kumar Panigrahy, Pinaki Samal

**Affiliations:** 1 Department of Livestock Production and Management, College of Veterinary Sciences and Animal Husbandry, Bhubaneswar - 751 003, Odisha, India; 2 Department of Animal Nutrition, ICAR - Central Institute for Women in Agriculture, Bhubaneswar - 751 003, Odisha, India; 3 Division of Livestock Production and Management, ICAR - National Dairy Research Institute, Karnal - 132 001, Haryana, India

**Keywords:** fermented fish silage, Japanese quail, proximate composition, serum biochemical parameters

## Abstract

**Aim:**

The objective of this study was to evaluate the effect of feeding fermented fish silage (FFS) on serum biochemical parameters of Japanese quails (*Coturnix coturnix japonica*).

**Materials and Methods:**

A total of 192, 7-day-old broiler Japanese quail chicks of either sex, were randomly distributed into four dietary treatments with four replicates in each group having 12 chicks in each replicate pen. The dietary treatments were T_1_ – Control diet, T_2_ – Diet containing 5% FFS, T_3_ – Diet containing 10% FFS, and T_4_ – Diet containing 15% FFS. Group body weight and feed consumption were recorded at weekly intervals. Feed conversion ratio (FCR) was derived by dividing the feed consumed with the weekly body weight gain. At the end of the experiment, 8 birds from each treatment were selected randomly and sacrificed by cervical dislocation to study the carcass traits expressed as % pre-slaughter live weight. At 5 weeks of age, about 2 ml of blood was taken from the jugular vein of each selected bird, and serum samples were separated after centrifugation. Total protein, albumin/globulin (A/G) ratio, calcium, phosphorus, triglyceride, total cholesterol, high density lipoprotein-cholesterol (HDL-C), very low density lipoprotein-cholesterol (VLDL-C), low density lipoprotein-cholesterol (LDL-C), alanine aminotransferase (ALT), and aspartate aminotransferase (AST) were estimated in the serum.

**Result:**

The cumulative body weight gains from 1 to 5 weeks of age did not vary significantly between control and 5% FFS group. However, a linear decrease in body weight gain was observed by increasing the levels of FFS beyond 5% in the diet. The cumulative feed consumption was significantly higher in case of birds under control group during 1-5 weeks of age as compared to birds fed FFS based diet (5%, 10%, and 15%). No statistical difference in cumulative feed conversion ratio could be noticed during 1-5 weeks of age. The eviscerated yield decreased and giblet weight increased due to the dietary supplementation of FFS at 15% level. The breast meat yield decreased due to dietary supplementation of FFS at 10% and 15% level in the diet of broiler Japanese quails. The effect of FFS was found to be nonsignificant (p>0.05) with respect to serum total protein, globulin and A/G ratio under different treatments whereas significant difference observed in serum albumin concentration. Dietary supplementation of FFS at 10% and 15% level significantly increased the AST concentration in serum. There was no significant difference among the treatments regarding the parameters such as ALT, calcium, phosphorous, triglyceride, total cholesterol, HDL-C, VLDL-C, and LDL-C.

**Conclusion:**

The serum-biochemical parameters are influenced by the dietary supplementation of FFS in broiler Japanese quails.

## Introduction

Poultry nutrition plays a key role on the performance, health and welfare of the animal and efficiency in feeding is one of the key factors for successful production. However, feed cost accounts around 65-70% of the total cost of production which is a growing challenge for the poultry sector. To overcome this challenge, some alternative protein sources like fermented fish silage (FFS) has been tested to identify the possibility of incorporation in poultry ration.

India is the second largest producer of fish in the world, contributing 5.68% of the global production. Fish processing for human consumption yields around 40% of edible meat while remnant 60% is fishery byproduct composed of bones, skin, head, viscera, meat scraps and scales [1] which are discarded as waste worldwide that causes serious environmental problems and economic losses [2,3]. Fish waste can be transformed into fish meal but the major pitfall being high cost of production and periodic scarcity [4]. FFS can be a promising alternative to fish meal as a source of cheaper and high-quality protein for animal feeding as it is environment friendly, safer, technologically simpler, and more economical than the manufacture of fish meal [5]. FFS is a liquid product made from whole fish or parts of it to which lactic acid producing bacteria or other fermentable carbohydrate sources are added, with the liquefaction of the mass provoked by the action of enzymes in the fish.

With increasing demand for fast food and competition among broiler and layer farmers, some alternative and equally competitive farming have also become very essential for the survival of the farmers. Quail farming can be a potential alternative to chicken farming because of its small size, delicacy, less floor space requirement, rapid growth, short generation interval, cardiac friendly, nutritious meat and egg production potentiality, short incubation period, less susceptibility to disease and low feed intake leading to its suitability for commercial rearing under intensive management [6-8]. Due to similarity to chicken in several aspects, Japanese quails have been used as pilot animal for pathological, nutritional, and physiological studies [9]. Blood biochemical profile - such as glucose, calcium, total protein, aspartate aminotransferase (AST), alanine aminotransferase (ALT), urea, and chloride levels - is of diagnostic values for various disease conditions and having particular reference to liver disorders, kidney diseases, diarrhea, dehydration, etc. [10]. Enzyme activity can be useful in evaluating the efficacy of fish silage as well as in selecting males to improve fertility and or hatchability of females in chicken [11].

Serum biochemical profile elicits nutritional and physiological status of an individual. The information regarding the effect of feeding FFS on the serum biochemical profile is scanty, especially in case of Japanese quails. Therefore, this study was undertaken to assess the dietary incorporation of FFS on the serum biochemical parameters of broiler Japanese quails (*C. coturnix japonica*).

## Materials and Methods

### Ethical approval

This study was duly approved by the Institutional Animal Ethics Committee, OUAT, College of Veterinary Science and Animal Husbandry, Bhubaneswar - 751 003, Odisha, India.

### Experimental program

The present biological trial was conducted at the Central Institute for Women in Agriculture (ICAR), Bhubaneswar for 4 weeks from the past week of March 2016 to mid of April 2016 during early to mid-summer.

A total of 192, 7-day-old, broiler Japanese quails of either sex were procured from the Central Poultry Development Organization, Bhubaneswar, and were randomly distributed into four treatment groups. There were four pens, each having a floor area of 3.72 m^2^ with 48 chicks in each pen, divided into four replicates in it. The dietary treatments were: T_1_ – Control diet; T_2_ – Diet containing 5% FFS, T_3_ – Diet containing 10% FFS, and T_4_ – Diet containing 15% FFS. All the diets were isocaloric (2900 kcal of ME/kg) and isonitrogenous (24% CP). Each diet was fed ad lib to one of the treatment group during the experimental period of 7-35 days of age.

### Preparation of FFS

Dressed waste (intestine and gills) of freshwater fishes were collected from a local fish market in Bhubaneswar, Odisha. The waste was washed in portable water, chopped and ground using meat grinder into paste for silage preparation. FFS was prepared by adding jaggery to the paste. 200 g (20%) jaggery was mixed with 1 kg fish silage. Addition of 200 ml water was done to make it more liquid. 200 ppm butylated hydroxytoluene (BHT) was added to prevent auto-oxidation and 1000 ppm potassium sorbate was added as mold inhibitor. 0.2 g BHT and 1 g potassium sorbate were mixed with 1 kg fish silage. Ensilation process was aided by incubating the materials in airtight plastic containers at room temperature (28-30°C). The silage was stirred twice daily to ensure the uniform distribution of jaggery. The fermentation process took about a week.

### Proximate principle analysis

The proximate analysis of FFS as well as the feed sample was conducted to evaluate the various parameters such as dry matter, total ash, ether extract, and protein content [12].

### Traits measured

Group body weight and feed consumption were recorded at weekly intervals. Feed conversion ratio (FCR) was derived by dividing the feed consumed with the weekly body weight gain. At the end of the experiment, 8 birds from each treatment were selected randomly and sacrificed by cervical dislocation to study the carcass traits and expressed as % pre-slaughter live weight.

### Collection of blood and analysis of serum sample

Two birds from each replicate were selected on random basis at the end of experiment (5 weeks of age). About 2 ml of blood was taken from the jugular vein of each selected bird by puncturing it with 24 gauze needle. Blood samples were collected in sterilized dry centrifuge tubes without any anticoagulant and then kept in room temperature for 2 h. After 2 h, the tubes were kept in incubator at 37°C for 30 min and then centrifuged at 3000 rpm for 15 min to separate the serum. The separated serum samples were kept in sterilized tubes marked and then preserved at −10°C for further analysis.

The serum biochemical parameters including total protein, albumin/globulin (A/G) ratio, calcium, phosphorous, total cholesterol, high density lipoprotein-cholesterol (HDL-C), very low density lipoprotein-cholesterol (VLDL-C), low density lipoprotein-cholesterol (LDL-C), triglyceride, ALT, and AST were estimated by following the procedures described in the reagent kit supplied by Span diagnostics limited, a product of ARKRAY Health Care Private limited, Surat, India, by using Biochemistry autoanalyzer.

### Mortality

Daily mortality of broiler Japanese quails was recorded in case of each treatment group. The mortality percentage of each treatment was calculated. The dead birds were then sent for autopsy to find out the cause of death and were disposed of by maintaining proper hygiene and sanitation.

### Statistical analysis

The data pertaining to various parameters were subjected to statistical analysis by using Statistical Package for Social Science version 17.0 under completely randomized design employing one-way analysis of variance. The means of different treatments were compared with Duncan’s multiple range test, and significance was considered at p<0.05 level [13,14].

## Results

The mortality of broiler Japanese quail chicks under different treatments is presented in Table-1. The mortality rate of broiler Japanese quails up to 5 weeks of age varied between 6.25% and 10.41% among the different dietary treatments. However, no effects of diets were noticed on the mortality of the birds.

**Table-1:** Mortality rate of broiler Japanese quails up to 5 weeks of age.

Mortality (%)	Treatments			

	T_1_ (control diet without FFS)	T_2_ (diet with 5% FFS)	T_3_ (diet with 10% FFS)	T_4_ (diet with 15% FFS)
	6.25 (3/48)	8.33 (4/48)	6.25 (3/48)	10.41 (5/48)

The figure in parenthesis represents number of birds dead/total number of birds. FFS=Fermented fish silage

In this experiment, dietary supplementation of FFS at 5% level did not result in growth depression at 5 weeks of age (Table-2). However, feeding of FFS at higher levels (10% and 15%) in the diet of Japanese quail chicks resulted in significantly (p<0.001) poor body weight compared to either control or 5% FFS. The cumulative body weight gains from 1 to 5 weeks of age did not vary significantly between control and 5% FFS group. However, a linear decrease in body weight gain was observed by increasing the levels of FFS beyond 5% in the diet (Control=5% FFS >10% FFS or, 15% FFS). Dietary supplementation of FFS reduced the feed consumption (Table-2) of broiler quail chicks, irrespective of the level of inclusion. The birds in the control group consumed significantly higher amount of feed during 1-5 weeks of age compared to birds fed FFS based diet (5%, 10%, and 15%). No statistical difference in cumulative FCR (CFCR) could be noticed during 1-5 weeks of age.

**Table-2:** Effect of feeding FFS on body weight gain, feed consumption and FCR of broiler Japanese quails at 1-5 weeks of age.

Parameters/treatments	Age in weeks	T_1_ (control diet without FFS)	T_2_ (diet with 5% FFS)	T_3_ (diet with 10% FFS)	T_4_ (diet with 15% FFS)	SEM	p value
Cumulative body weight gain (g)	1-5	123.59^c^	125.33^c^	112.41^b^	86.39^a^	2.869	0.001
Cumulative feed consumption (g)	1-5	439.84^d^	392.14^c^	347.35^b^	326.93^a^	11.391	0.001
Cumulative FCR	1-5	3.56	3.13	3.09	3.78	1.551	0.001

a-d Mean bearing different superscripts in a row differ significantly (p<0.05). FCR=Feed conversion ratio, FFS=Fermented fish silage, SEM=Standard error of mean

The carcass characteristics of broiler Japanese quails under different treatments are presented in Table-3. The eviscerated weight did not vary significantly due to dietary supplementation of FFS up to 10%, but significantly lower eviscerated weight was observed in the 15% FFS group compared to control (T_1_). The relative weight of giblet (liver + heart + gizzard) increased significantly in the 15% FFS dietary group compared to control. The broiler quail chicks under T_1_ group (without FFS) have been recorded similar breast meat yield (23.86±0.372) to that of 5% FFS group (23.22±0.372) which was significantly higher than other dietary groups which did not vary significantly among each other (T_1_=T_2_>T_3_=T_4_).

**Table-3:** Effect of feeding FFS on carcass yield of broiler Japanese quails at 5 weeks of age.

Parameters/treatments	T_1_ (control diet without FFS)	T_2_ (diet with 5% FFS)	T_3_ (diet with 10% FFS)	T_4_ (diet with 15% FFS)	SEM	p value
Eviscerated yield	62.01^b^	60.18^ab^	61.53^ab^	59.77^a^	0.350	0.066
Giblet*	5.51^a^	5.76^a^	5.47^a^	6.59^b^	0.135	0.006
Breast meat	23.86^b^	23.22^b^	21.53^a^	20.99^a^	0.372	0.007

a,b Mean bearing different superscripts in a row differ significantly (p<0.05),

* Giblet (liver+heart+gizzard). SEM=Standard error of mean, FFS=Fermented fish silage

The serum biochemical profiles of broiler Japanese quails under different treatments are presented in Tables-4-7.

**Table-4:** Effect of dietary supplementation of FFS on serum protein profile (g/dl) of broiler Japanese quails at 5 weeks of age.

Parameters	T_1_ (control diet without FFS)	T_2_ (diet with 5% FFS)	T_3_ (diet with 10% FFS)	T_4_ (diet with 15% FFS)	SEM	p value
Total protein (g/dl)	5.76	5.48	5.23	5.72	0.162	0.655
Albumin (g/dl)	2.49^b^	2.34^b^	2.09^a^	2.42^b^	0.044	0.002
Globulin (g/dl)	3.27	3.13	3.14	3.30	0.158	0.977
A/G ratio	0.87	0.79	0.68	0.76	0.049	0.652

a,b Mean bearing different superscripts in a row differ significantly. SEM=Standard error of mean, FFS=Fermented fish silage

**Table-5:** Effect of dietary supplementation of FFS on serum lipid profile (mg/dl) of broiler Japanese quails at 5 weeks of age.

Parameters	T_1_ (control diet without FFS)	T_2_ (diet with 5% FFS)	T_3_ (diet with 10% FFS)	T_4_ (diet with 15% FFS)	SEM	p value
Triglyceride (mg/dl)	121.23	110.60	108.84	120.18	1.02	0.458
Total cholesterol (mg/dl)	144.79	134.38	149.85	137.37	6.781	0.866
HDL-C (mg/dl)	89.22	89.10	94.50	91.56	1.24	0.340
VLDL-C (mg/dl)	31.33	23.16	33.59	21.78	2.05	0.209
LDL-C (mg/dl)	24.24	22.12	21.76	24.03	0.85	0.640

SEM=Standard error of mean, FFS=Fermented fish silage, HDL-C=High density lipoproteincholesterol, VLDL-C=Very low density lipoproteincholesterol, LDL-C=Low density lipoproteincholesterol

**Table-6:** Effect of dietary supplementation of FFS on serum mineral profile (mg/dl) of broiler Japanese quails at 5 weeks of age.

Parameters	T_1_ (control diet without FFS)	T_2_ (diet with 5% FFS)	T_3_ (diet with 10% FFS)	T_4_ (diet with 15% FFS)	SEM	P value
Calcium (mg/dl)	9.32	9.12	8.97	10.18	0.203	0.147
Phosphorous (mg/dl)	6.74	6.94	6.69	7.53	0.336	0.828

SEM=Standard error of mean, FFS=Fermented fish silage

**Table-7:** Effect of dietary supplementation of FFS on serum enzymatic profile (mg/dl) of broiler Japanese quails at 5 weeks of age.

Parameters	T_1_ (control diet without FFS)	T_2_ (diet with 5% FFS)	T_3_ (diet with 10% FFS)	T_4_ (diet with 15% FFS)	SEM	p value
AST (IU/L)	84.97^a^	97.70^a^	122.70^b^	143.30^b^	6.110	0.001
ALT (IU/L)	30.65	31.55	30.90	30.05	0.685	0.901

a,b Mean bearing different superscripts in a row differ significantly. SEM=Standard error of mean, ALT=Alanine aminotransferase, AST=Aspartate aminotransferase

The serum total protein in the blood of broiler Japanese quails at 5 weeks of age ranged from 5.23 (T_3_) to 5.76 g/dl (T_1_) under different treatments. The effect of supplementation of FFS in the diet of broiler Japanese quails was found to be nonsignificant (p>0.05) with respect to serum total protein, globulin and A/G ratio under different treatments. Lowest albumin concentration in serum was noticed in the 10% FFS group which was significantly different from other dietary groups.

The serum calcium and phosphorous concentrations in the blood of broiler quails ranged from 8.97 to 10.18 mg/dl and 6.69 to 7.53 mg/dl, respectively. There was no significant difference in the serum calcium and phosphorous content in different treatments.

Dietary supplementation of FFS at 5% level had no influence on serum AST concentration, but higher levels of FFS (10% and 15%) significantly increased the AST concentration in serum. No difference could be observed in the serum ALT concentration across the dietary groups.

There was no significant difference among the treatments regarding the parameters such as triglyceride, total cholesterol, HDL-C, VLDL-C, and LDL-C.

## Discussion

The mortality of quail chicks under different treatments was within the normal range indicating that dietary supplementation of FFS up to 15% in the diet of broiler Japanese quails had no adverse effect on the health condition of the quail chicks. The variation in mortality among the different dietary treatments might be due to summer stress and cannot be attributed to treatment effect. The present findings were in agreement with the reports of Boitai who observed low mortality for all dietary treatments [15].

The variation in the level of supplementation of FFS in Japanese quails could be attributed due to their adaptive capacity and species variation. The cumulative body weight gains from 1 to 5 weeks of age did not vary significantly between control and 5% FFS group. However, a linear decrease in body weight gain was observed by increasing the levels of FFS beyond 5% in the diet. From this finding, it is inferred that FFS at 5% can be included in the diet of broiler Japanese quails level without affecting the body weight gain. In contrast to this, Al-Marzooqi *et al*. reported significant increase in body weight and body weight gain by feeding fish silage at the rate of 10% and 20% [16]. The cumulative feed consumption was significantly higher in control group of birds as compared to the birds under FFS-based dietary groups (5%, 10% and 15% FFS). No statistical difference in CFCR could be noticed during 1-5 weeks of age. This finding of this study revealed that quail chicks can efficiently utilize the FFS even up to 15% in the diet. This is in agreement with the findings of Ramirez *et al*. who observed no difference in FCR due to inclusion of fish silage in the diet of broiler Japanese quails [4].

The data on carcass characteristics (Table-3) observed in this study were within the normal range found in Japanese quails. The eviscerated yield decreased and giblet weight increased due to the dietary supplementation of FFS at 15% level. In contrast to this, Boitai reported no adverse effect of dietary inclusion of 10% acid treated fish silage on eviscerated yield of broiler chickens [15]. The breast meat yield decreased due to dietary inclusion of FFS at 10% and 15% level in the diet of broiler quail chicks. From the results of this study, it can be inferred that FFS at 10% and 15% level in the diet of broiler quail chicks had an adverse effect on dressed weight as well as breast meat.

The analyzed values of the different serum biochemical parameters were reported to be within the normal range [17]. The non-difference with respect to the total protein concentration in the serum indicated that dietary supplementation of FFS up to a level of 15% was adequate to meet the normal protein requirements of broiler Japanese quails. The albumin concentration in serum among the groups varied significantly; the control group being the highest which was contrary to Boitai in which the control was having a lesser amount of albumin than the treatment groups [15].

The non-difference in serum triglycerides, total cholesterol, and different cholesterol fractions (HDL, VLDL, and LDL) due to the feeding of 15% FFS in broiler quail chicks implied that dietary supplementation of FFS up to 15% had no adverse effect on triglycerides and cholesterol fractions in the serum [18].

The different macro and micro minerals form the structural components of the body, which participate in the proper functioning of body systems. The calcium and phosphorous concentration in the serum did not vary significantly across the dietary treatments, indicating that 15% FFS in the diet exhibited adequate minerals to meet the requirements as well as to maintain the normal mineral metabolism of the broiler quail chicks [17].

No difference was observed in the ALT concentration in the serum [19]. The AST activity in serum was significantly higher in the diet containing 10% and 15% FFS compared to control and 5% FFS based dietary groups. Since major activity of transaminase is seen in liver, any condition leading to large-scale degeneration of cells would result in liberation of these enzymes into the circulating blood stream [20]. 15% FFS might have increased the metabolic activity of liver cells which might have synthesized and degraded more of dispensable amino acids leading to higher concentration of AST enzymes in the serum. In this study, relatively higher liver weight was observed in the 15% FFS based dietary group, further adding to higher metabolic activity of liver leading to hypertrophy of liver.

## Conclusion

The serum biochemical values of broiler Japanese quails were analyzed as influenced by the dietary supplementation of FFS. The results indicated that the protein profile, lipid profile, and mineral profile along with ALT was not influenced due to supplementation of FFS in the diet of quail chicks. Dietary supplementation of more than 5% FFS (either 10% or 15%) significantly increased the AST activity in the serum. Supplementation of 5% FFS in the diet of broiler Japanese quails indicated that they satisfy the nutritional requirements of Japanese quails without affecting the performance and this practice would also help the fish industry to increase their income and provide a safe methodology to mitigate pollution generated from fish waste. Further study could also be carried out to investigate the application of this procedure in the nutritional regime of other livestock species.

## Authors’ Contributions

SP, LKB and AKP designed the plan of work. SP and AKP conducted the experiment. SP, AM and PS performed laboratory investigations. AM, TS, PS and KKP helped in the laboratory investigations. SP, LKB, AKP and KKP participated in draft and revision of the manuscript. All authors read and approved the final manuscript.
